# Primed and cued: long-term acoustic telemetry links interannual and seasonal variations in freshwater flows to the spawning migrations of Common Snook in the Florida Everglades

**DOI:** 10.1186/s40462-022-00350-5

**Published:** 2022-11-13

**Authors:** Jordan A. Massie, Rolando O. Santos, Ryan J. Rezek, W. Ryan James, Natasha M. Viadero, Ross E. Boucek, David A. Blewett, Alexis A. Trotter, Philip W. Stevens, Jennifer S. Rehage

**Affiliations:** 1 Institute of Environment, Department of Earth and Environment, Florida International University, 11200 SW 8th St., Miami, FL USA; 2 Institute of Environment, Department of Biological Sciences, Florida International University, Miami, FL USA; 3 Department of Marine Science, Coastal Carolina University, Conway, SC USA; 4 Bonefish & Tarpon Trust, Florida Keys Initiative, Marathon, FL USA; 5 Florida Fish and Wildlife Conservation Commission, Fish and Wildlife Research Institute, Port Charlotte, FL USA; 6 Florida Fish and Wildlife Conservation Commission, Fish and Wildlife Research Institute, St. Petersburg, FL USA

**Keywords:** Acoustic telemetry, *Centropomus undecimalis*, Coastal fishes, Freshwater flow, Migratory drivers, Movement ecology, Long-term data, Partial migration, Riverine fishes, Skipped spawning

## Abstract

**Background:**

Spawning migrations are a widespread phenomenon among fishes, often occurring in response to environmental conditions prompting movement into reproductive habitats (migratory cues). However, for many species, individual fish may choose not to migrate, and research suggests that conditions preceding the spawning season (migratory primers) may influence this decision. Few studies have provided empirical descriptions of these prior conditions, partly due to a lack of long-term data allowing for robust multi-year comparisons. To investigate how primers and cues interact to shape the spawning migrations of coastal fishes, we use acoustic telemetry data from Common Snook (*Centropomus undecimalis*) in Everglades National Park, Florida, USA. A contingent of Snook migrate between rivers and coastal spawning sites, varying annually in both the proportion of the population that migrates and the timing of migration within the spawning season. However, the specific environmental factors that serve as migratory primers and cues remain unknown.

**Methods:**

We used eight years of acoustic telemetry data (2012–2019) from 173 tagged Common Snook to investigate how primers and cues influence migratory patterns at different temporal scales. We hypothesize that (1) interannual differences in hydrologic conditions preceding the spawning season contribute to the number of individuals migrating each year, and (2) specific environmental cues trigger the timing of migrations during the spawning season. We used GLMMs to model both the annual and seasonal migratory response in relation to flow characteristics (water level, rate of change in water level), other hydrologic/abiotic conditions (temperature, salinity), fish size, and phenological cues independent of riverine conditions (photoperiod, lunar cycle).

**Results:**

We found that the extent of minimum marsh water level prior to migration and fish size influence the proportion of Snook migrating each year, and that high river water level and daily rates of change serve as primary cues triggering migration timing.

**Conclusion:**

Our findings illustrate how spawning migrations are shaped by environmental factors acting at different temporal scales and emphasize the importance of long-term movement data in understanding these patterns. Research providing mechanistic descriptions of conditions that promote migration and reproduction can help inform management decisions aimed at conserving ecologically and economically important species.

**Supplementary Information:**

The online version contains supplementary material available at 10.1186/s40462-022-00350-5.

## Background

Migration is a widespread phenomenon occurring in animal populations worldwide, with examples spanning diverse habitats, in taxa ranging from terrestrial insects to marine mammals, and on scales varying from small displacements to thousands of kilometers [1–4]. Because of the diversity of organisms and contexts in which migration occurs, definitions in the literature are varied [5]. However, common themes emerge. Migration entails the collective directional movements of individuals or groups between well-defined and spatially distinct habitats, which provide favorable ecological conditions for a period of time, and frequently occurs on a cyclical or recurrent basis [4–6]. Migrations take place over a spectrum of environmental conditions and are motivated by factors related to changing resource dependencies, physiological needs, and/or seeking refuge to avoid unfavorable conditions [7–9].

Migratory patterns arise from a complex suite of genetic, physiological, behavioral, and ecological factors that are ultimately driven by the optimization of growth, survival, and reproduction [9, 10]. However, the timing and pathways of migration often occur in response to proximate *cues* related to seasonality and changes in environmental conditions [11]. Despite the increase in attention on migratory cues, research remains limited in the number/types of species investigated [9, 12]. Further, there is a need for research that addresses not only migratory cues but also how environmental conditions at broader timescales (e.g., conditions experienced in the months leading up to migration) may serve as migratory *primers*, influencing the decision to undertake reproductive migrations each year [13, 14]. Inference on migratory primers has been limited in part by a lack of long-term data that can help quantify the relative importance of environmental drivers in contributing to interannual differences in migration and associated reproduction [15–17].

Reproductive migrations commonly span environmental gradients (e.g., aquatic/terrestrial, salinity regimes), and adults move into habitats providing appropriate environmental and physiological requirements for successful breeding and development of offspring [9, 18]. For coastal fishes, seasonal fluctuations in water level dictate several key processes that may influence the spatial-temporal spawning landscape. The magnitude, duration, and abruptness of change in freshwater flows can alter productivity gradients, physicochemical environments, and tidal and current flows, all of which can influence reproductive success and recruitment [19–22]. As such, variation in freshwater flows can serve as cues for reproductive migrations.

In rivers, changes in flow have been shown to trigger spawning migrations in multiple species including Estuary Perch [*Macquaria colonorum*, 23], Australian Bass [*Macquaria novemaculeata*, 24], Australian Grayling [*Prototroctes maraena*, 15], Mary River Cod [*Maccullochella mariensis*, 25], Mulloway [*Argyrosomus japonicus*, 26], European Eel [*Anguilla anguilla*, 27], and Barramundi [*Lates calcarifer*, 28, 29]. Acoustic telemetry studies have indicated that both the probability and scale of migratory movements increase with river discharge [23, 24, 30]. Taylor et al. [26] reported that high flows drove the riverine migrations of Mulloway, potentially serving as a signal promoting the formation of spawning aggregations in the lower estuary. However, variation in migratory timing has been reported. For Australian Bass and Australian Grayling, individuals initiated migrations at different times and on distinct flow pulses during a spawning season [15, 24]. Further, the directionality and magnitude of flow may influence the strength of the migratory responses. For example, large-scale movements of neotropical prochilodontids (*Prochilodus costatus*) and large catfish (*Phractocephalus hemioliopterus*, *Pseudoplatystoma punctifer*) in Brazilian rivers were detected during the transition between dry periods and rising water levels [31, 32], and migrations of catadromous eels in New Zealand (*Anguilla* spp.) corresponded to days with increasing discharge [33].

**Fig. 1:**
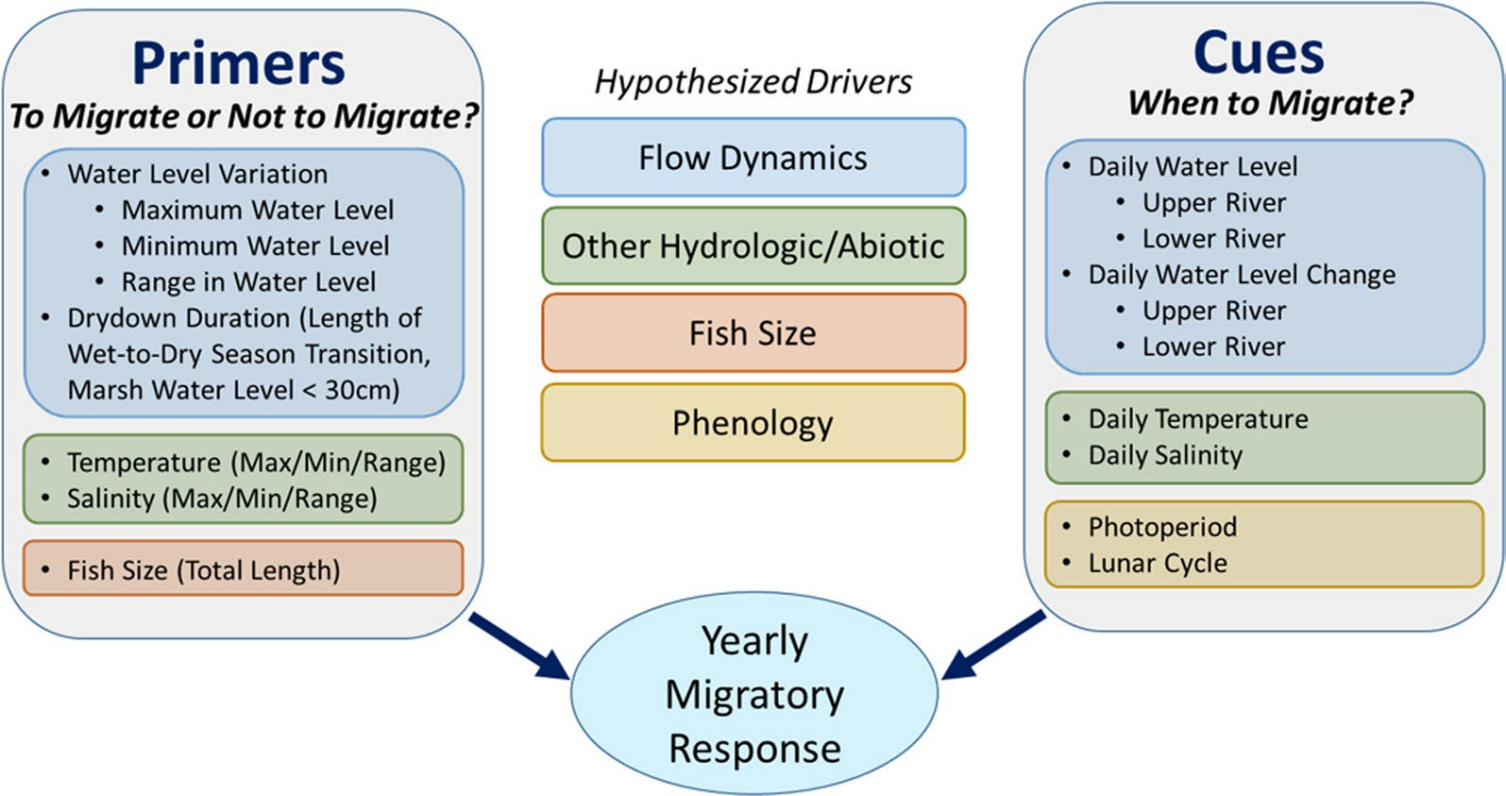
Conceptual and analytical framework to investigate the environmental drivers of Common Snook migration at multiple temporal scales. We hypothesize that migration results from a combination of pre-spawning environmental conditions influencing the proportion of fish that migrate (primers), and environmental cues that determine the timing of migrations within a spawning season

A recurring observation in studies of spawning migrations is a high degree of interannual variability in migratory behaviors [24, 32, 34]. Relatively few studies have focused on how conditions experienced before the reproductive season (migratory primers) may influence the extent of partial migration for species that forgo spawning in a given year, often referred to as skipped spawning in fishes [9, 14, 35]. In some cases, the proportion of individuals migrating each year (hereafter referred to as intensity of the migratory response) has been linked to variability in precipitation, which could both affect the relative strength of migratory cues and result in differences in juvenile survival [32, 36]. Other studies have suggested that energetics may play an important role in the decision to migrate, with evidence for lower energy reserves increasing the prevalence of skipped spawning [13, 14]. Whether or not fish respond to environmental cues and initiate a spawning migration may be dependent on conditions experienced months earlier, with annual migration patterns reflecting the interaction between both *primers* and *cues* acting at different temporal scales. Here we propose an organizational framework that is both conceptual and analytical for addressing the primers and cues of spawning migrations (Fig. 1), and takes advantage of long-term acoustic telemetry data and the unprecedented understanding of migration patterns it provides [37].

Common Snook (*Centropomus undecimalis*, hereafter Snook) are a tropical euryhaline fish species well-suited to studies of migration. Snook are found in freshwater and marine habitats of the western Atlantic, the Caribbean, and the Gulf of Mexico, with their range extending from Brazil to Cedar Key on Florida’s gulf coast and Cape Canaveral on the Atlantic coast [38–40]. The species has received considerable research attention due to their recreational and economic importance [39, 41]. While some fish reside in lower estuaries and marine waters [42, 43], a migratory portion of the population lives in riverine habitats for much of the year [34, 38, 44, 45]. Freshwater prey serve as an important seasonal resource for migrant Snook, and upriver movements into freshwater habitats correspond to drying marshes when prey are concentrated in river channels in advance of the spawning season [38, 46–49]. Snook are marine obligate spawners and require high salinity for successful reproduction [50–52]. Both estuarine and riverine contingents must move to the lower estuary and ocean inlets to spawn. In Florida, spawning occurs over a protracted period beginning in April and extending through November [43, 53–57]. Downstream migrations from freshwater habitats to coastal spawning sites have been previously documented by acoustic telemetry and indicate a high degree of variability in migratory behaviors [34, 43, 58, 59]. Not all Snook migrate each year. Studies from both the Atlantic and gulf coasts have estimated skipped spawning ranging from 24 to 40% [34, 58, 59]. No previous work has tied Snook migrations to specific environmental factors, and there remains a need for quantitative descriptions of the mechanisms and conditions that drive migratory behaviors to help inform fisheries management [38, 60–62].

In this study, we use eight years of acoustic telemetry data (2012–2019) to examine how environmental variability at both annual and seasonal scales influence the spawning migrations of Snook in Everglades National Park (ENP), Florida, USA. More specifically, our goal was to investigate the pre-spawning conditions that maximize the migratory responses and promote reproduction (primers) and dictate the intensity of migration, as well as the daily cues during the spawning season that act to initiate migrations as a function of hydrologic variation, and thus drive the timing of such migrations. Our research questions are twofold: Q1) how does interannual variation in hydrologic conditions influence the intensity of the migratory response each year, and do certain conditions act as primers for population-level migratory responses? (Fig. 1 —To migrate or not to migrate?), and Q2) do specific environmental cues trigger the timing of Snook migration within a given season? (Fig. 1 —When to migrate?). We hypothesized that: H1) interannual differences in hydrologic conditions preceding the spawning season, and associated variation in the timing/extent of transitions between the wet and dry season that drives access to prey pre-spawning, contribute to the intensity of the Snook migratory response, and H2) specific cues presented by changes in hydrologic conditions within the spawning season trigger the timing of Snook migrations (e.g., high flow events, temperature, salinity). To test these hypotheses and understand the intensity and timing of spawning migrations, we modeled the migratory response of Snook in relation to a suite of environmental variables. We selected explanatory variables previously reported to influence migration and reproduction in fishes [15, 16, 20, 23, 63].

## Methods

### Study area

**Fig. 2:**
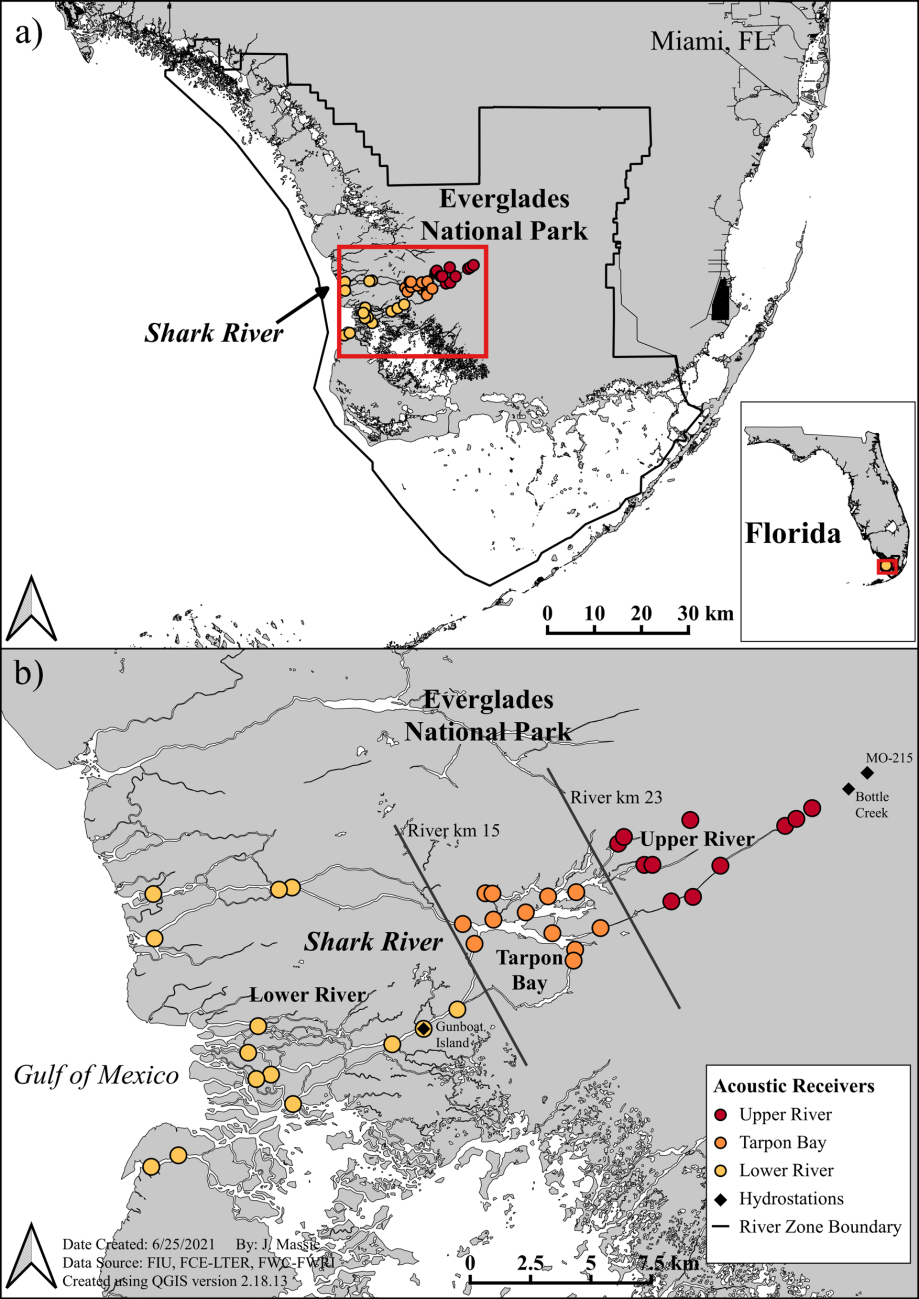
Map of the study area in Everglades National Park. Panel (**a**) shows the location of the Shark River in SW Florida, and panel (**b**) depicts the configuration of the acoustic array used to monitor the movements of Common Snook. Black lines indicate delineation between different river zones used to identify migrations of Common Snook (upper river, Tarpon Bay, lower river), colored circles indicate the placement of acoustic receivers and river zone designation, and black diamonds show the location of hydrologic monitoring stations where environmental conditions were measured

The Shark River is an expansive, low-gradient coastal river system in the southwestern region of ENP that extends 32 km inland with a drainage area encompassing roughly 1700 km^2^ (Fig. 2). The hydrologic regime is shaped by a subtropical climate and seasonal freshwater flows which are driven by tidal cycles and rainfall patterns influenced by atmospheric teleconnections on both short (El Niño/Southern Oscillation) and long (Atlantic Multidecadal Oscillation, hereafter AMO) timescales, resulting in variability in both the timing and the total amount of precipitation and annual flow characteristics (Additional file 1: Fig. S1; [64–66]). Alterations to the natural hydrology have occurred over the last century due to drainage and impoundment for urban and agricultural development that have reduced the volume of freshwater entering the system [67]. Paleo-based estimates indicate that historic flow levels were 2.1 times greater than those currently found [68, 69]. Despite these changes, the historic wet/dry seasonal pattern has been retained, and > 75% of the system’s rainfall occurs during the wet season in May through October, with a dry season of November to April [65, 66, 70, 71].

The headwaters of the Shark River consist of small creeks and marshes that transition into mangrove forests, with progressively larger and more saline channels approaching the coast at the Gulf of Mexico [65, 66, 72]. The system can be broadly divided into three zones with distinct habitat characteristics [46, 49, 73, 74]. The oligohaline upper river (salinity range 0–5 PSU) receives limited tidal influence and consists of shallow narrow channels bordered by a combination of mangroves and freshwater marshes [47, 75–77]. The mesohaline mid-river (salinity range 1–21 PSU) is characterized by a shallow open embayment (Tarpon Bay) with larger mangrove forests, and receives more pronounced daily tidal fluctuations relative to the upper river [47, 48]. The polyhaline lower river is the most tidally-influenced, with salinities ranging seasonally from 10 to 36 PSU between the wet and dry seasons [77] and contains the most productive mangrove forest of the Everglades [78].

### Acoustic telemetry

Acoustic monitoring of Snook movements in the Shark River began in January 2012 as part of the Florida Coastal Everglades Long Term Ecological Research program [67]. Fish were captured monthly via boat-based electrofishing along shoreline habitats at 15 sites in the upper Shark River and Tarpon Bay [methods detailed in 47]. Upon capture, adult Snook were placed in an aerated livewell and total length (TL, mm) and weight (whole, g) were recorded. Sex was not assigned to captured individuals. Electrofishing was primarily conducted outside of the spawning season when external indicators of sex (e.g., milt produced when pressure applied, visible oviduct opening posterior to anal slit) that are present in mature and actively spawning fish are less apparent [59, 79, 80]. Fish were then transferred to an onboard tagging station within 2–3 min of capture and held ventral side up in a v-shaped cradle with the head and gills submerged. Implantation of acoustic tags followed the methods of Young et al. [45, 59] which were adapted from the procedures outlined by Lowerre-Barbieri et al. [43] and have been shown to minimize stress and maximize survival for the species. Tagging consisted of a minor surgical procedure where a 30 mm incision was made in the lower abdomen and an acoustic transmitter (Vemco 69 kHz V13 or V16, Innovasea, Halifax, NS, Canada) inserted into the abdominal cavity, and the incision closed with a single Vicryl™ suture. Following tagging, fish were held in water alongside the boat and allowed to regain equilibrium before release. The estimated battery life for acoustic tags was 3.7 years (1,349 days) or 6.7 years (2,435 days) for V13 and V16 tags respectively.

Between 2012 and 2019, tagged Snook were continuously monitored by an array of 37 Vemco VR2W acoustic receivers (Innovasea, Halifax, NS, Canada) placed 1–3 km apart in a gated design, allowing us to track directional movements throughout the Shark River (Fig. 2b). Each monitoring station was assigned a river distance reflecting its position relative to the coast (river km), which ranged from 0 km at the Gulf of Mexico to 32 km in the headwaters. Unique detections for individual fish were associated with a date, time, and river km within the array. Past research in the Shark River has demonstrated the efficacy of this deployment configuration for quantifying fish movement and changes in distribution over time [46, 49, 74, 81].

### Identifying migrations

To determine if and when a fish migrated, we considered acoustic detections for each individual in each spawning season they were detected. Telemetry data were screened prior to analysis and fish with less than 10 unique detections in their movement histories were excluded from analysis. This screening process allowed us to identify unreliable observations (false detections) consisting of single detections that could not be confirmed on more than one receiver, and only include fish with a sufficient record to provide inference into migratory movements [23, 59, 82]. Fish were considered migrants if they were recorded making directed downstream movements from the upper river or Tarpon Bay into the lower river towards coastal receivers during the spawning season (Fig. 2b). If an individual moved downstream and was detected on at least one lower river receiver followed by either the end of their detection history or a time gap until subsequent re-detection in the lower river, they were presumed to have moved to coastal spawning sites. While portions of the lower river may reach salinities required for the buoyancy of fertilized eggs [> 24 PSU, 43, 45], Snook spawning has been shown to take place at ocean inlets and coastal marine areas in other Florida populations [43, 45, 59], and we would expect Shark River fish to use similar marine spawning habitats. Although coastal spawning activity could not be directly confirmed, past studies have shown how movements from freshwater/estuarine to marine areas correspond to Snook spawning activity, suggesting that downstream migration during the spawning season is indicative of spawning activity [59, 79]. For our analyses, we defined the spawning season as April 1 to November 15, a window consistent with previous observations of Snook spawning activity in Florida [46, 83]. Migration timing for each individual was recorded as the year and date during the spawning season when the initiation of downstream migration occurred, which was then related to hypothesized drivers of migration using statistical models. For fish that moved persistently downstream after initiating migration, migration timing was assigned as the date that the individual entered the lower river. If an individual was detected migrating over the course of several days, migration timing was recorded as both the date of directed movement from the upper river and into Tarpon Bay, and the date of movement from Tarpon Bay into the lower river.

### Environmental data: primers and cues

To examine possible drivers of Snook migration, we modeled a suite of environmental covariates in relation to migratory responses from our telemetry data at both annual and seasonal scales (Fig. 1, Additional file 1: Tables S1 and S2). Environmental data consisted of flow metrics (water level, daily water level change) and key hydrologic/abiotic variables (temperature, salinity) reported to influence movement and migration in fishes [15, 20, 24, 32, 81, 84, 85]. We also included variables examining a potential phenological response (photoperiod, lunar cycle) independent of hydrologic variation. Daily time series data for mean daily water level relative to NAVD 88 were obtained from the Everglades Depth Estimation Network (EDEN, https://sofia.usgs.gov/eden/). Water level data from two different monitoring stations in the Shark River were initially considered (Fig. 2b, Bottle Creek in the upper river, Gunboat Island in the lower river) but these measurements were collinear (Pearson correlation 0.7), and exploratory models indicated better model fit using data from the upper river. Thus, the upper river monitoring station (Bottle Creek) was selected to represent water level in our final models. We also considered river discharge as a candidate flow metric, but it was highly collinear with water level (Pearson correlation 0.9). Using water level improved model performance relative to discharge, and thus water level was selected as a representative variable for flow conditions. Water level from an additional monitoring station (Fig. 2b, MO215) located in the freshwater marsh adjacent to the upper river was also included to quantify the wet/dry seasonal transition period (drydown duration), a period of prey concentration in the river channels during receding water levels [46–48]. Daily temperature and salinity data were queried from the United States Geological Survey time-series for Bottle Creek (Station 022908295) via the South Florida Water Management District’s environmental database (DBHYDRO, https://www.sfwmd.gov/science-data/dbhydro). To examine whether cumulative environmental change may better explain migratory movements relative to daily variation, we evaluated model performance of hydrologic variables (water level, water level change, temperature, salinity) for daily mean, 3-day mean, and 7-day mean data. In all cases daily mean data resulted in the best model performance. Thus, daily mean observations were selected for use in our final models.

### Modeling annual primers: to migrate or not to migrate?

To test our hypothesis that conditions prior to the spawning season act as primers influencing the intensity of the migratory response at an interannual scale, we performed logistic regression using generalized linear mixed models (GLMMs) with a binomial error distribution and unique acoustic tag numbers for each fish as a random effect. The response variable was a binary indicator for each individual and year that noted whether a fish migrated (1) or did not migrate (0) during that year. Analyses were performed in R statistical software [86] using the glmmTMB package [87].

Modeling was performed in a four-step process where we first examined all candidate variables that characterized each hypothesized driver (Fig. 1, Additional file 1: Table S1). Second, when collinearity was found among variables, Akaike’s information criterion [AIC, 88] was used to select the best fitting variable. Third, the selected variables were then combined into a global model. And fourth, backward selection was performed using the step() function from the stats package in R [86] to select a final model based on the lowest AIC [89–92]. For each of our models, fit was also assessed by relative model weight and by calculating R-squared values showing the amount of model variance explained using the Performance package in R [93].

We examined a set of hypothesized drivers to explain interannual migration patterns and act as migratory primers in the months prior to migration (flow dynamics, other hydrologic/abiotic conditions), along with the role of fish size in the migratory response (Additional file 1: Table S1). Variables for migratory primers captured riverine conditions found during the preceding dry season (152-day period from November 15 in the prior year to April 1 of the current year, end of the previous spawning season to the beginning of the current spawning season). This period was selected based both on its role in the sexual maturation of Snook, and as a period shown to provide enhanced opportunities for foraging on freshwater prey as water levels drops through the dry season [38, 46, 47]. Snook are protandrous hermaphrodites, transitioning from mature males to females at sizes that range from a total length (TL) of 264 to 876 mm [57, 94]. Histological analysis of female Snook indicated that the months between spawning periods correspond to the development and regeneration of oocytes, and high hepato-somatic indices suggest that sex transition and maturation is occurring outside of the spawning season [57]. Because gametogenesis and reproductive migrations are energetically costly, resource acquisition during this period can be particularly important. Results from Young et al. [57] support capital breeding to some extent for Snook, and that energy derived outside of the spawning season is used during reproduction [95]. Because the timing of peak prey concentration can vary widely from year-to-year based on the annual hydrograph (Additional file 1: Fig. S1), we included primer variables based on the full period from the end of the previous spawning season to the beginning of the current spawning season to allow for the broadest set of environmental variation. Exploratory models also included factors characterizing the annual hydrologic conditions occurring within each spawning season (April 1–Nov 15), but they showed only weak correlations and did not improve model performance. Thus, only water level and hydrologic/abiotic variables from the dry season preceding the spawning season were included in our final models.

We considered primer metrics representing the dry season maximum, minimum, and overall range of water levels, temperature, and salinity. Due to the importance of seasonal freshwater prey subsidies for Snook [38, 46, 47], we calculated a primer metric quantifying the duration of potential high-quality foraging opportunities in the upper river leading into the spawning season. This metric (drydown duration) reflected the total number of days the freshwater marshes adjacent to the river dropped below 30 cm in the dry season, a water level that has been found to correspond to increases in abundance/biomass of marsh prey seeking refuge in the creeks and channels of the upper Shark River (R. Rezek, unpublished data).

The probability of migration for Snook has been reported to increase with fish size [63, 96], and scale samples were initially collected during tagging to determine age and estimate future growth. However, laboratory analyses conducted by the Florida Fish and Wildlife Conservation Commission have shown scales to be an unreliable method for ageing Snook [94]. Scale derived estimates consistently underestimated ages determined by otolith analysis by up to three years for fish younger than 10 years old, the period during which the most rapid growth has been recorded for the species [94]. In order to provide insight on the role of size in Snook migration, we followed the methods of Young et al. [59] and estimated a total length (TL) for each fish at the beginning of each spawning season. These length-age estimates were based on von Bertalanffy growth curves derived from otolith analysis of 7970 Snook collected as part of a fishery-independent monitoring program on Florida’s gulf coast, and are reported in stock assessments for the State of Florida [39, 94]. We first calculated an estimated age for each fish based on TL at the time of tagging, then projected growth to the beginning of each spawning season in which that individual was detected. Fish age at tagging was calculated using parameters from Taylor et al. [94] and the equation:

$$t=\frac{1}{K}\text{ln}\left(\frac{L-{L}_{t}}{L}\right)+{t}_{0}$$

where *t* = age of fish when tagged, *K* = growth coefficient for gulf coast Snook, *L* = asymptotic length, *L*_*t*_ = length at time of capture, and *t*_*0*_ = hypothetical age for a fish at length zero. A “current” age was assigned as the estimated tagging age plus the time between tagging and the beginning of a new spawning season. The above equation was then transformed and solved to determine fish size in each subsequent year of detection (new *L*_*t*_) as follows:

$${L}_{t}= -1*\left(L\left(\text{exp}\left(K\left(t- {t}_{0}\right)\right)-L\right)\right)$$

### Modeling seasonal cues: when to migrate?

To identify environmental factors that influence migration timing for Snook within a spawning season, we used a second set of binomial GLMMs to test our hypothesis that specific cues, namely changes in hydrologic conditions (water level, water level change, temperature, salinity), trigger the timing of migration. Here, the response was a binary variable (1/0) for each individual and detection day indicating the timing of downstream migration during spawning season (April 1–Nov 15). A response value of 1 indicated that an individual Snook had initiated a directed downstream movement into the lower river zone from either the upper river or Tarpon Bay on that day, and a 0 value was assigned to days where no migratory movements were detected. Because the focus of these models was to identify cues prompting migratory behaviors, only fish detected making a downstream migration were included in analysis. Further, migratory status (0 or 1) was only assigned to days where fish were detected on the array in order to not draw inference where data was not available. As with our models of annual migration primers, we first assessed the best variable or variables for the hypothesized driver. Second, we removed lower fit collinear variables based on AIC. Our process consisted of an additional step compared to the annual models, and we then considered a combination of variables within each hypothesized driver (Fig. 1, flow dynamics, other hydrologic/abiotic conditions, phenology) in order to examine the relative role of that driver in predicting migration probability. We used AIC to select either the best fitting variable or combination of variables for each hypothesized driver, and then combined all best fitting variables into a global model. Last, we reduced this global model using backward selection. A final model best able to explain the timing of Snook migrations was selected based on the lowest AIC, relative model weight, and amount of variance explained.

Our models for seasonal cues consisted of variables that evaluated the relationships between migratory timing and hypothesized environmental drivers quantifying flow, other hydrologic/abiotic conditions, and phenological cues (Fig. 1, Additional file 1: Table S2). For flow, in addition to mean daily water level, we included a variable for the daily water level change to test our hypothesis that changes in flow serve as an important migratory cue [32, 33]. We considered absolute water level change in early models, but this resulted in poor model performance relative to daily water level change, which differentiates between increases and decreases in water level. Mean daily temperature and salinity were selected to represent other important hydrologic/abiotic conditions that vary throughout the spawning season and could influence migratory timing, consistent with the hypothesized drivers from our annual primer models. We also included variables examining whether migration was influenced by environmental factors independent of in-river conditions. Both photoperiod and lunar cycle were used to indicate the presence of a fixed phenological migratory response to seasonality. Further, a variable for the year was included to assess the role of interannual variability in Snook migration, capture additional variance operating at annual scale but not related to other fitted variables, and match our annual primer models.

## Results

### Migration patterns: intensity of spawning migration

Over the course of the study, 206 individual Snook were tagged ranging in size from 416 to 1,010 mm TL (mean 690  ± 139 mm). The number of fish tagged per year ranged from 14 to 55 (mean 26). A total of 189 individuals were subsequently detected on the array after release (92% of all tagged fish). Of these, two individuals did not meet our detection criteria to provide inference on migratory movements (minimum of 10 unique detections in movement history) and were excluded from further analysis. One of these fish had only a single detection indicating a possible false detection, and data for the second fish contained a total of nine detections, all occurring within a 24-hour period. An additional fourteen fish had no detections occurring within any of the eight focal spawning seasons (between April 1 and November 15, 2012–2019).

**Fig. 3:**
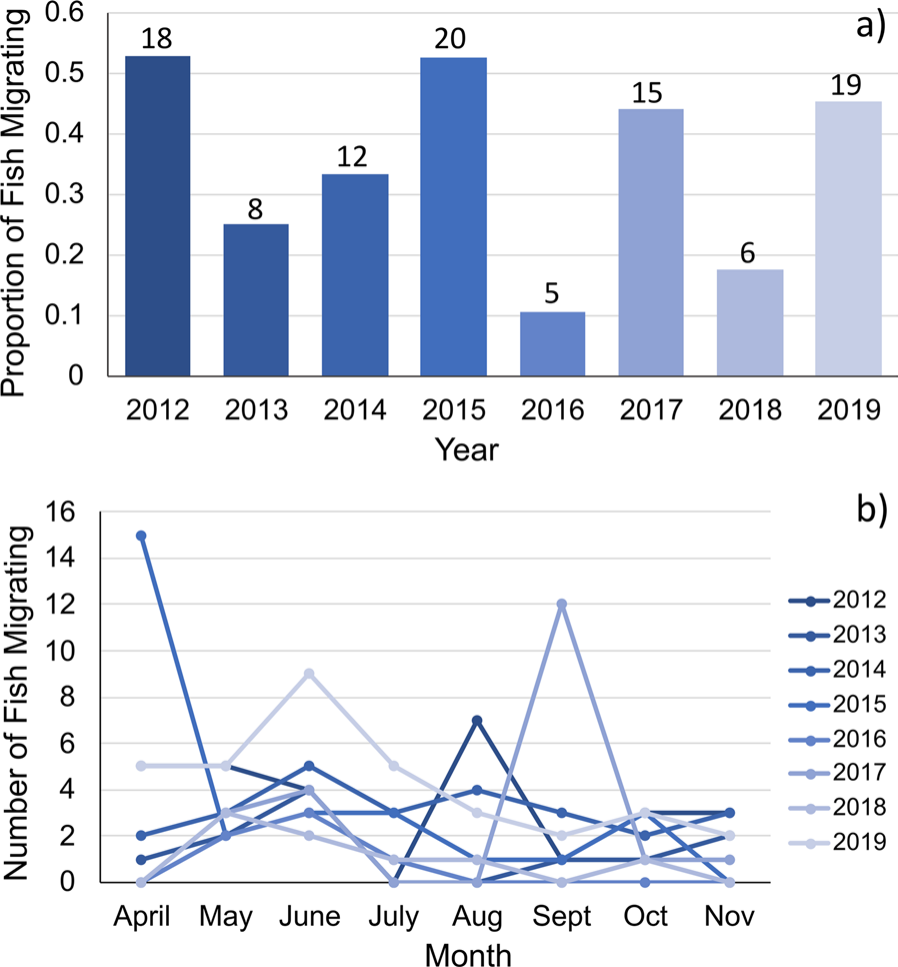
Illustration of the high degree of interannual and seasonal variability in both the proportion of tagged Common Snook migrating each year, and the timing of migration within each spawning season. Panel (**a**) depicts the proportion of fish observed migrating in each year of the study, ranging from 11% to 2016 to 53% in 2012 and 2015, with the total number of individual migrants detected each year noted above each bar. Panel (**b**) illustrates the protracted migration period, with migrations occurring in all months of the spawning season. Each year is color coded and consistent between panels (**a**) and (**b**). See Additional file 1: Tables S3 and S4 for additional information

In total, 173 Snook were detected in at least one spawning season. Of these fish, 90 individuals (52%) were detected making downstream migrations during the spawning season, with some fish detected migrating in multiple years. Of the migrants, 78 Snook were detected migrating in only a single spawning season, 11 individuals were detected migrating in two seasons, and one fish was detected migrating in three seasons. When accounting for detections in multiple years, 297 unique fish/year combinations (migrants and non-migrants) served as the basis for our annual primer models, with 103 observed migrations included in our seasonal cues models (Additional file 1: Table S3). The proportion of fish detected migrating in a single year (number of migrants / total number of fish detected each year) ranged from a high of 53% in 2012 and 2015 to a low of 11% in 2016, with a mean annual migration rate of 35% of detected individuals between 2012 and 2019 (Fig. 3a).

### Migration patterns: timing of spawning migration

Snook initiated downstream migrations in all months of the spawning season. The temporal migration patterns varied strongly from year to year, but over the course of the study more fish migrated in April, May, and June than any other month, representing > 50% of all detected migrations (Fig. 3b, Additional file 1: Table S4). The greatest number of fish were detected migrating in June (21%), and the fewest in November (7%). May and June were the only two months where migrations were observed in each of the 8 years of the study.

**Fig. 4:**
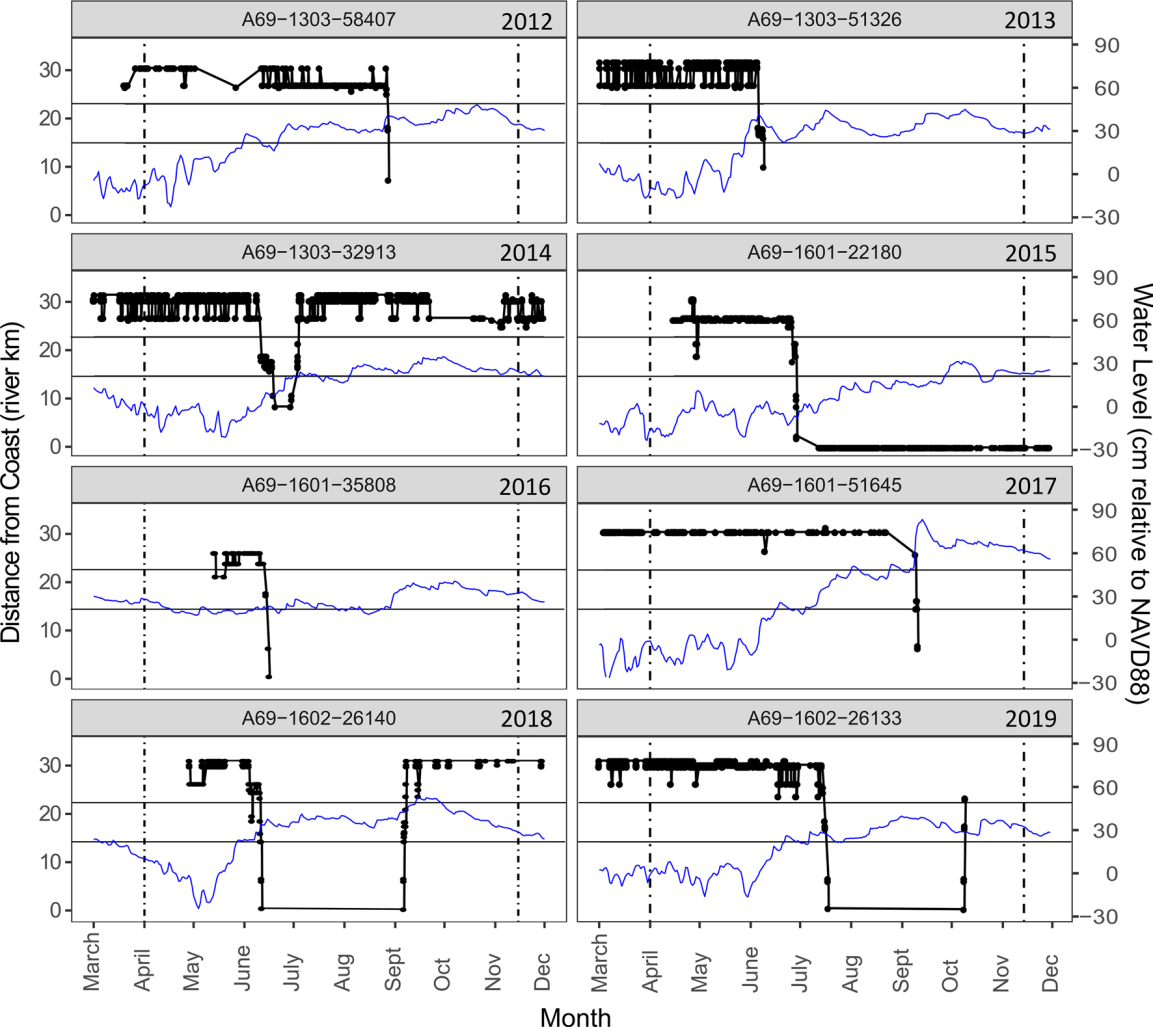
Examples of individual movement tracks from acoustic detections of tagged Common Snook (fish ID number noted in each panel) showing downstream migrations during the spawning season. Solid blue lines show the measured water level at Bottle Creek in the upper river for each year. Horizontal black lines at river km 23 and 15 delineate zones boundaries between the upper river (> 23 river km), Tarpon Bay (15–23 river km), and the lower river (< 15 river km, per Fig. 1), and vertical hashed lines mark the beginning and end of the spawning season (April 1-Nov 15)

We found two predominant movement types in migrating Snook. Slightly less than half of the migrants (43%) moved continuously from the upper river to the coast after initiating migration and were detected on the downstream-most receivers within 48 h of departure (examples in years 2012, 2015–2019 of Fig. 4). The other individuals made rapid and directed downstream movements from the upper river to Tarpon Bay, paused, and were then detected on receivers within the bay for between 2 days and 1 week before continuing their migration into the lower river (57% of migrants, depicted in years 2013 and 2014 in Fig. 4).

### Annual primer models: to migrate or not to migrate?

**Fig. 5:**
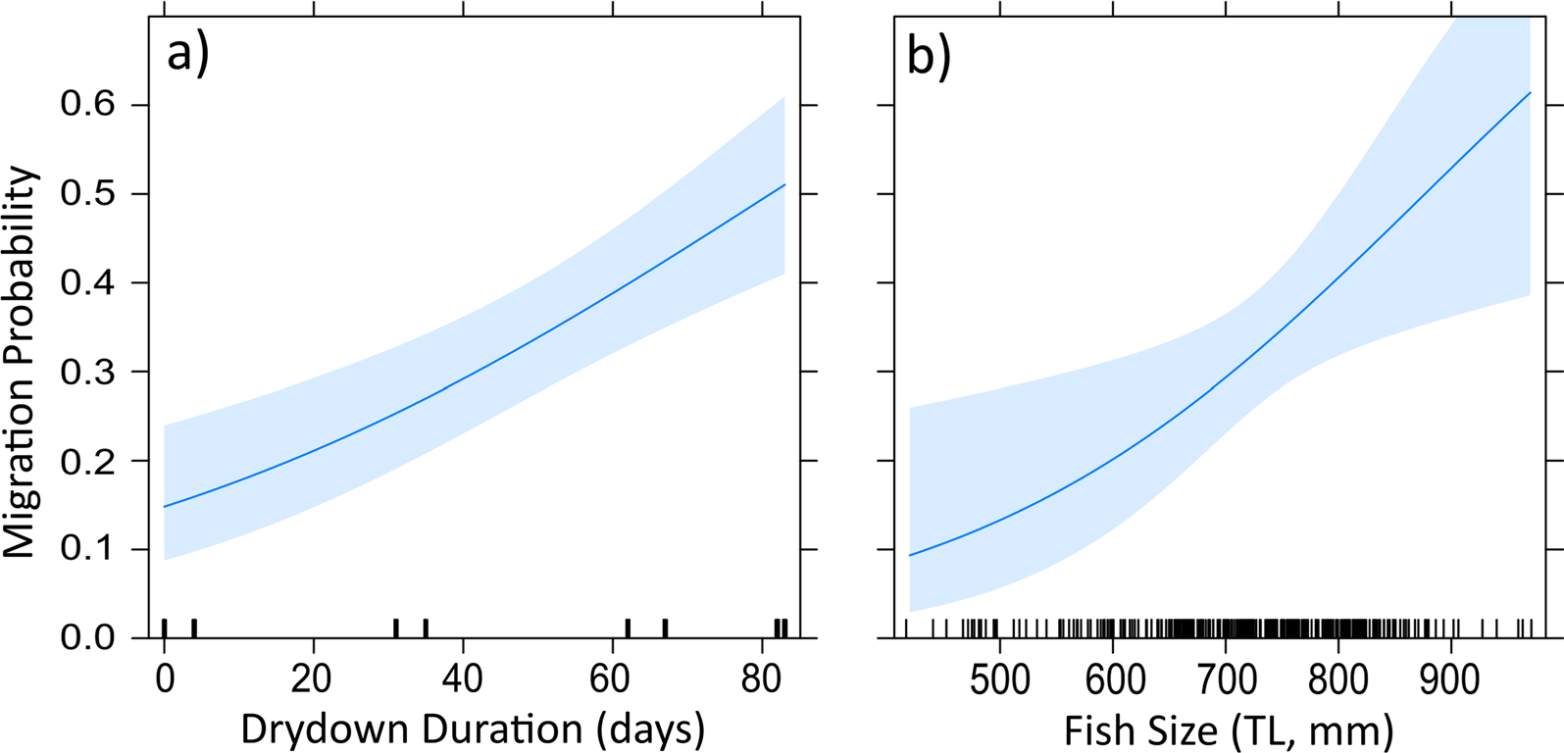
Plotted variables for the best-fitting logistic regression model (Drydown Duration + Fish Size) for the annual migratory intensity of Common Snook bounded by a 95% confidence interval. Individual effects of each variable kept in the best model in Table 1 are assessed by holding the other variables at a fixed mean value. Together these variables explain 27.6% of the variability in the proportion of tagged fish migrating each year

After applying our 4-step model fitting protocol, five variables were selected for our global model (Table 1). After performing backward selection, the final model consisted of only two variables, drydown duration and fish size. This best model explained a comparable amount of model variance relative to the more complex global model (R-squared 0.276 and 0.299 respectively), but with a lower AIC score. Comparisons of all models indicated a substantially higher weighting for the reduced model (AIC weight = 0.9466) relative to any univariate model or the global model. A univariate model for drydown duration explained > 20% of model variance, outperforming all other variables. Both predictors from the final model indicated a significant positive relationship to annual migration probability (Table 2), with the proportion of fish migrating increasing with both drydown duration and fish size (Fig. 5).

**Table 1:** Model selection results based on lowest AIC value from GLMM models examining the drivers of annual migratory intensity (migratory primers) for Common Snook

Hypothesized driver	Model variables	df ^†^	AIC	ΔAIC ^‡^	AIC Weight	Conditional R^2^	Marginal R^2^
Flow dynamics	Minimum water level	3	364.3	13	0.001	0.175	0.122
	Drydown duration	3	359.4	8.1	0.017	0.204	0.135
Other hydrologic/abiotic conditions	Maximum temperature	3	379	27.7	< 0.001	0.079	0.048
	Salinity range	3	369.7	18.4	< 0.001	0.147	0.083
Fish size	Fish size	3	372.6	21.3	< 0.001	0.151	0.085
Global model	Minimum water level + drydown duration + maximum temperature + salinity range + fish size	10	357.9	6.6	0.035	0.299	0.234
Best model	Drydown duration + fish size	4	351.3	0	0.947	0.276	0.197

The response variable for models is whether each individual fish was detected migrating or not migrating each year, across all eight years of tracking. For variable descriptions see Additional file 1: Table S1

†Model degrees of freedom; ‡ Difference in AIC score between each model and lowest AIC model

**Table 2:** Summary statistics for the best GLMM model predicting the intensity of annual Common Snook migration (see Table 1)

Variable	Beta	SE	z-value	*p *value
(Intercept)	− 5.333	1.472	− 3.622	< 0.01
Drydown duration	0.022	0.005	4.371	< 0.01
Fish size	0.005	0.002	2.593	< 0.01

Results show a positive significant relationship between the proportion of fish migrating annually and the length of the transitional marsh drydown period prior to the spawning season (drydown duration) plus fish size

### Seasonal cues models: when to migrate?

After assessing the individual and combined effects of variables for each of our hypothesized drivers affecting the timing of Snook migration, six variables were selected for the global model (Table 3). High values for collinearity (Pearson coefficient > 0.06) were not present for any variable pairings, thus variable selection for the global model was based on AIC. For flow, the best model (lowest AIC) was a combination of both water level and daily water level change. A combined model was selected for additional hydrologic/abiotic cues and included both temperature and salinity. For the variables representing a phenological response, we selected the model containing only lunar cycle. AIC was within 2 points of a combined model also including photoperiod, although photoperiod was not statistically significant (p = 0.09). Therefore, we chose the simpler model to combine into a global model. Additionally, a variable for the year of migration was included in the global model.

**Table 3:** Model selection results based on lowest AIC value from GLMM models examining the environmental cues for Common Snook migration during the spawning season

Hypothesized driver	Model variables	df^†^	AIC	ΔAIC^‡^	AIC Weight	Conditional R^2^	Marginal R^2^
Flow dynamics	Water level	3	2082	144.2	< 0.001	0.265	0.119
	Daily water level change	3	2051	112.8	< 0.001	0.119	0.086
	Water level + daily water level change	4	1980	41.5	< 0.001	0.292	0.175
Other hydrologic/abiotic conditions	Temperature	3	2174	235.3	< 0.001	0.042	0.006
	Salinity	3	2171	233.1	< 0.001	0.042	0.006
	Temperature + salinity	4	2169	230.6	< 0.001	0.039	0.012
Phenology	Day length	3	2176	237.6	< 0.001	0.053	0.003
	Lunar cycle	3	2175	236.3	< 0.001	0.051	0.005
	Day length + Lunar cycle	4	2174	235.6	< 0.001	0.058	0.008
Interannual variation	Year	9	2174	235.9	< 0.001	0.061	0.024
Global model	Water level + daily water level change + temperature + salinity + lunar cycle + year	14	1938	0	0.680	0.319	0.247
Best model	Water level + daily water level change + temperature + salinity + year	13	1940	1.6	0.320	0.316	0.245

The response variable is a binary indicator of whether or not each fish was detected migrating for each day detected. For variable descriptions see Additional file 1: Table S2

†Model degrees of freedom; ‡ Difference in AIC score between each model and lowest AIC model

**Fig. 6:**
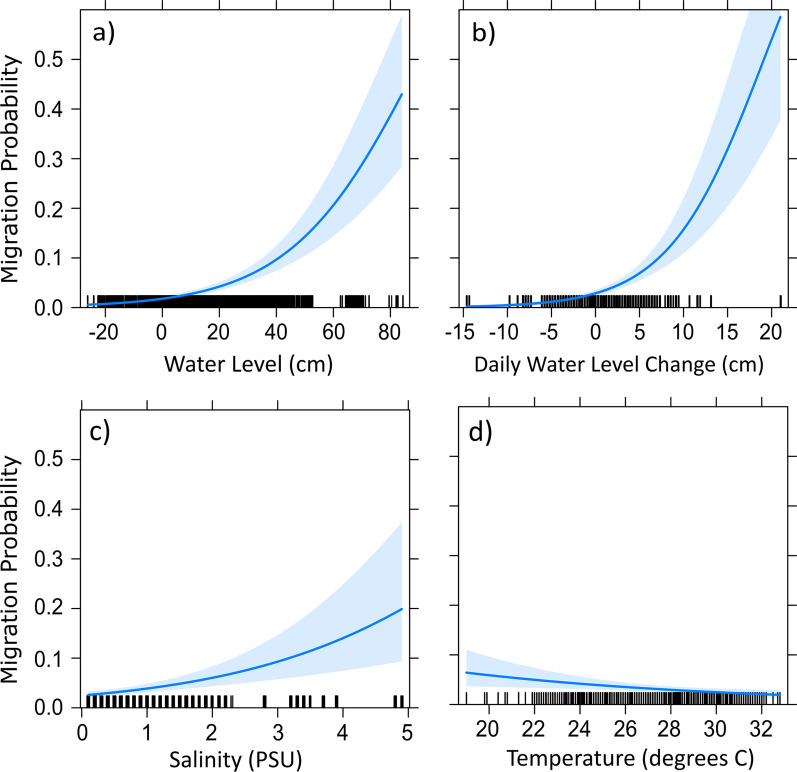
Plotted variables for the best-fitting logistic regression model for the daily environmental cues predicting downstream migration timing for Common Snook during the spawning season bounded by a 95% confidence interval. Individual effects of each variable kept in the best model shown in Table 3 are assessed by holding the other variables at a fixed mean value. Together these variables explain 31.6% of the variability in the timing of migration within the spawning season. Water levels in panels (**a**) and (**b**) reflect gauge height relative to NAVD 88 from the Bottle Creek monitoring station, and salinity and temperature in panels (**c**) and (**d**) reflect daily mean measurements at Bottle Creek

When these variables were included as covariates and reduced by backward selection, the best model included all variables except lunar cycle (Table 3). These two models were within 2 AIC points of each other indicating comparable performance; however, lunar cycle was not significant in the model (*p* = 0.06) and both models explained nearly the same amount of model variance (R-squared ~ 0.32 for both models). Thus, the simpler reduced model containing covariates for water level, daily water level change, temperature, salinity, and year was selected as our best model (Table 3). A large proportion of the model variance was explained by the variables for water level and daily water level change, which accounted for > 29% of variance. In the final model, coefficients for water level, daily water level change, and salinity showed a significant positive relationship with the probability of migration, whereas temperature showed a weak negative correlation (Table 4). These results indicate that the probability of Snook initiating downstream migrations during the spawning season increases with water level, daily water level change, and salinity, and migration probability decreases with temperature (Fig. 6).

**Table 4:** Summary statistics for the best GLMM model (see Table 3) predicting the timing of Snook migration relative to daily environmental cues

Variable	Beta	SE	z-value	*p *value
(Intercept)	− 1.483	0.901	− 1.647	0.10
Water Level	0.044	0.005	9.338	< 0.01
Daily water level change	0.184	0.021	8.568	< 0.01
Temperature	− 0.090	0.033	− 2.724	< 0.01
Salinity	0.466	0.102	4.571	< 0.01

Results show a positive significant relationship between the probability of fish initiating downstream migration and the daily water level, water level change, and salinity, and a negative relationship with temperature

## Discussion

In this study, we investigated how environmental variability at multiple temporal scales affects spawning migrations, and how annual primers which maximize migratory intensity (the proportion of the population that migrates each year) and seasonal cues that trigger migration timing interact to explain the interannual variation in the migratory response. Our results illustrate the complexity of migratory behaviors, and that decisions to migrate or not are influenced by a combination of factors that differ from those that affect when to migrate. We found correlations between Snook spawning migrations and hydrologic patterns at both interannual and seasonal scales. The proportion of Snook migrating increased in years with a longer drydown duration (specifically, the number of days with marsh water levels below 30 cm) before the spawning season, which we hypothesize is associated with enhanced feeding opportunities for floodplain prey [38, 46, 47] and results in increased energy reserves that promote migration/spawning. This suggests that water levels preceding the spawning season are an important primer for migration in the Shark River, influencing the population-level response and driving year-to-year variability in spawning, whereas water level and positive rates of change in water level (i.e., high flow events) cue the timing of migratory movements. These results add to a growing body of evidence that seasonality in the flow regime and river/floodplain dynamics are a central factor in the behavior and ecology of many coastal fishes [20–22, 97–99], yet provide an unusually detailed understanding of the dependency of spawning migrations on hydrological drivers, by pairing long-term tracking and environmental data.

While we documented a high degree of interannual and seasonal variability in the migration pattern of acoustically tagged Snook and its dependency on hydrological drivers, we recognize limits to the interpretation of our results. First, a small proportion of the population was tagged, and although samples sizes were large and adequate for multiyear analyses, they may or may not be representative of the migration patterns of the entire population. Second, because our study consisted of multiyear data and drew inference from movement patterns occurring months/years after tagging (i.e., a downriver movement to the coast constituted a spawning migration), we were not able to assess the reproductive status of tagged fish migrating each year. Moreover, we did not directly measure Snook activity along the coast where spawning aggregations may occur and were unable to confirm spawning for migrating fish. However, previous studies have shown that movements from rivers to marine areas correspond to reproductive readiness (e.g., oocyte development or postovulatory follicle sampling), suggesting that downstream migration during the spawning season can be used to indicate spawning activity [57, 79]. Further, Snook are protandrous hermaphrodites, and some skipped spawning may be related to lower energy reserves in newly transitioned females [57]. We were not able to sex tagged fish, and some individuals likely transitioned from male to female over the study period. Sex has been shown to influence spawning patterns and behaviors in Snook [59], and future work able to incorporate sex into models of migratory probability would offer valuable insight. We also acknowledge that cumulative hydrologic variation occurring at timescales longer than the daily changes included in our models could contribute to migratory timing (i.e., lagged effects). However, using daily data consistently resulted in the best model performance (lower AIC), suggesting the importance of discrete environmental cues in triggering migrations. Further, hydrological alterations affect many rivers inhabited by Snook, and these alterations may mute the environmental dependencies observed in the Shark River. Last, previous work has documented the presence of multiple contingents in Florida Snook, including riverine, coastal, and offshore marine segments of the population [42, 43], and we acknowledge that the marked dependency of spawning on freshwater flows may only apply to riverine Snook.

The presence of distinct spawning groups has been observed in other migratory fishes. Secor et al. [100] reported staggered migration timing for Striped Bass (*Morone saxatilis*) in the Hudson River, USA. Further, Koster et al. [15] found differences in migration timing for Australian Grayling. Distinct groups of tagged fish responded to separate high flow events within a single spawning season, and there was considerable variation in the proportion of Australian Grayling migrating each year (18–85% of tagged fish detected migrating annually). Differences in migration timing may be an adaptive strategy to account for environmental variation and temporal differences in spawning success, resulting in a portfolio effect that provisions for greater population stability over time [101].

Our results indicate a protracted spawning period for Snook, with migrations occurring in all months of the spawning season, consistent with patterns previously reported for the species [34, 43, 58, 59]. Yet, most of the spawning migrations were observed in earlier months (> 50% migrated April-June). The decision to spawn earlier or later in the season may offer distinct advantages for Snook. Earlier spawn times may provide juveniles a longer summer growing season where growth rates have been reported to be twofold higher relative to colder winter periods [1 cm/day vs. 0.5 cm/day, 54]. Predation risk declines with size for juvenile Snook, and increased growth may enhance overall survival for fish spawned earlier in the season [102]. However, late-season spawning provides greater access to flooded nursery habitats offering protection from predators, including adult Snook that have not yet migrated and may cannibalize juveniles [103].

### Annual primers: to migrate or not to migrate?

Our results suggest a high frequency of skipped spawning in tagged Snook in the Shark River. Skipped spawning has been well documented in Florida Snook populations, although the proportion varies among systems and years [34, 57, 58]. Our annual frequencies of between 11 and 53% of Snook migrating are in line with the annual migration/skipped spawning frequencies previously reported (24–40%) but suggest a higher degree of interannual variation in the Shark River [34, 57, 58]. This may be partly explained by the longer timespan of our data which encapsulates a broader range of hydrologic variation relative to previous studies. Similar patterns of skipped spawning have also been described for Striped Bass [104], prochilodontids [32], and Australian Bass [24]. Our findings suggest that the extent of skipped spawning may be related to environmental primers in advance of the spawning season which influence migration, namely marsh drydown duration. Further, we found that fish size positively correlated with migration probability. This relationship to size has been previously reported for Snook, and for other migratory species including Barramundi in Australia and Striped Bass in Chesapeake Bay [58, 59, 100, 105]. Both drydown duration, which affects access to freshwater prey resources, and fish size can indicate available energy resources, suggesting that energy status may serves as key factor in the decision to migrate each year.

Skipped spawning, rather than an anomaly, appears to be quite common among fishes and may serve as an adaptive behavior that can help maximize lifetime reproductive potential and mitigate for environmental variability [9, 13, 106]. Models using data from a wide range of migratory taxa indicated that skipped migrations were associated with environmental stochasticity, and the increased risk of a bad year leading to lowered fecundity and poor recruitment [35]. This hypothesis may be supported by the considerable variation in hydrological conditions in the Shark River (Additional file 1: Fig. S1), including droughts (2015) and prolonged flooding (2016, 2018). A study of Arctic Haddock (*Melanogrammus aeglefinus*) proposed that skipped spawning was related to body condition, and that lower energy reserves increased the probability of skipping [14]. For Snook, access to freshwater prey originating in floodplain marshes provides an important resource in the months prior to spawning [38, 47, 48, 107]. Our models indicate that drydown duration was a key variable explaining the annual proportion of Snook migrants. In the Shark River, hydrological variation is common and results from large-scale climatic events such as flooding/droughts accompanying El Niño and AMO cycles, from tropical storms, and from freshwater management decisions [64, 70]. This variation can influence both the timing and extent of the marsh prey pulse entering the river channels. In our data, years with the highest migration had the longest marsh drydown. Years with exceptionally low migration (2016, 2018) dried only briefly prior to the spawning season, and/or showed little seasonal variation in water level. We hypothesize that this drydown duration represents access to the freshwater prey pulse established by past research [38, 46, 47]. We suspect that a gradual prolonged drydown with higher prey concentrations in the river channels directly increases Snook energy reserves, resulting in higher proportions of migrants. Conversely, in years where the marshes dry only briefly, or not at all, available energy reserves are lowered and increase the prevalence of skipped spawning. Future work aims to better link this dry season prey pulse to Snook body condition and spawning activity.

### Seasonal cues: when to migrate?

Variation in river flow (both water level and daily water level change) was a primary cue driving the downstream migration timing of Snook. This is consistent with other studies of riverine fishes. Prochilodontids and large catfish in Brazil, long-lived eel species in New Zealand, Barramundi in Australia, and Australian Grayling undertake long-distance river migrations to spawning grounds at the onset of the rainy season, and like our findings, positive increases in water level were reported to trigger the initiation of migratory movements [15, 20, 29–33]. Harding et al. [24] also reported that high flows serve as migratory cues for Australian Bass, noting that not all fish responded to the same flow cue and multiple high flow events during the spawning season may increase the total number of individuals migrating. A migratory response to flow cues may serve as an adaptive behavior whereby elevated water levels signal the availability of high-quality juvenile rearing areas. In Australian rivers, for example, the growth rates of juvenile Barramundi increase with access to floodplain-derived resources which only become available during episodic and relatively short-duration seasonal flooding, yet account for a substantial portion (30–40%) of their diet [28, 99].

Salinity and temperature contributed to predicting the Snook migratory response in our models but explained only a small portion of model variance relative to water level and daily water level change. Temperature has been linked to the spawning activity of migratory fishes in other systems, but its relative role as a migratory cue varies. Legett et al. [16] reported that water temperature was the most consistent predictor of migratory abundance of River Herring (*Alosa* spp.) in Massachusetts. Daily increases in spring water temperature corresponded to increases in migratory activity. Conversely, Secor et al. [100] examined temperature as a driver of Striped Bass migration in the Hudson River, but concluded that while temperature did influence run timing (migration activity increased with increasing spring water temperatures), it was not tightly coupled with the migratory response and spawning was detected over a wide range of temperatures.

In Florida, Common Snook exist at the northern extent of their range and low winter water temperatures (< 10 C) can be lethal [40, 46, 108]. As a tropical species, maximum water temperature currently presents less of a threat to Florida Snook but may have some influence on reproductive timing and range expansion as waters warm under climate change. The upper thermal limits have been reported at approximately 35–42 C, with thermal preferences ranging from 26 to 29 C [109, 110]. Taylor et al. [56] noted that the reproductive season corresponds to periods where water temperatures are maximal, which could influence the timing of spawning for Snook. Roberts et al. [111] also suggested that an interaction between temperature and day length may play a role in stimulating gametogenesis and maturation in Snook. Conversely, Hernández-Vidal [112] did not find a strong relationship between temperature and gonadal development in Snook. However, low temperatures indicative of non-spawning periods were associated with lower levels of sex hormones, perhaps signaling end of spawning season.

### Importance to management

Annual variation in skipped spawning frequencies resulting from differences in hydrologic conditions could affect estimates of the reproductive potential of fish populations in stock assessments. For example, the spawning potential ratio (SPR, the total number of adult fish that remain in the population after accounting for harvest) is used by fisheries managers to represent reproductive potential in a given year [113, 114]. SPR may become highly variable if a large portion of the population does not reproduce annually. Skipped spawning is not currently incorporated into Snook stock assessments in Florida [39], and an improved understanding of how temporal and spatial variations affect skipped spawning would allow SPR estimates to be calibrated based on hydrologic conditions. While our models were not assessed for predictive performance, our results illustrate a high degree of interannual variability in skipped spawning, suggesting that this variation may be appropriate to consider in future estimates of reproductive potential for Snook and other species that undertake reproductive migrations.

Findings from this study could also provide water managers information that informs controlled freshwater releases that provision for ecological function in regulated systems. For example, Koster et al. [30] showed that Australian Grayling migrate downstream during both increasing and decreasing discharge surrounding high flow events, but that fish ceased migration once water levels stabilized, indicating that short duration flow releases may not be sufficient for complete migration. Harding et al. [24] also noted a similar pattern for Australian Bass; while many fish undertook uninterrupted downstream migrations to spawning sites during increased flow, some individuals moved only part of the way downstream and required multiple high flow events to navigate barriers and reach downstream spawning grounds. Thus, increasing the number of high flow events in a single season could maximize the total number of spawning fish. We observed similar patterns in our tagged Snook, with roughly half of the migrating fish demonstrating staged migrations consisting of rapid directed movements from the upper river to Tarpon Bay, followed by a second movement event to the coast separated by days/weeks.

Quantitative descriptions of flow requirements that promote migration, reproduction, and recruitment could be used to provide conditions that enhance fisheries production. For instance, environmental flows designed to promote the migration and spawning of Australian Grayling have been informed and adapted by research revealing key flow characteristics [15]. Flow patterns initially set to trigger spawning activity were adjusted to also include cues promoting migration. In South Florida, flow patterns and water releases are highly managed for urban uses, agriculture, and flood control. Managed freshwater releases that incorporate both seasonal drawdowns maintaining access to important prey resources, coupled with pulsed increases in water level triggering migration during the spawning season, can assist in providing both the primers and cues influencing the spawning migrations of Snook.

Our study, though informative, accounts for only 27.6% of the variance in interannual migration frequency and 31.6% of the variance in migratory timing. However, there are areas which could help explain additional model variance. Energy status can play a key role in the decision to migrate [13, 14], and future research could expand on previous work by explicitly linking temporally variable prey landscapes and energy status to migration. Another emerging direction in studies of migration is how social cues and interactions may affect migration [115]. In many cases, migration can be a collective decision and migrating as a group can assist in navigation, conserve energy through schooling behaviors, and provide safety in numbers from predators [6, 116, 117]. For example, Furey et al. [118, 119] described how the migratory success of salmon smolts was tied to outmigration density and increased survival when high numbers increased the ability of individual fish to evade predation. Future research should focus on the role of energy status, social cues, and density dependence to provide additional insight on migratory behaviors to assist in fisheries management.

## Conclusion

We provide evidence of how flow patterns influence migratory behavior at multiple temporal scales, serving as both environmental primers promoting the intensity of the migratory responses and seasonal cues that influence the timing of spawning migrations. Importantly, our results emphasize the critical role of long-term movement data, which can reveal patterns not apparent in shorter-duration studies and provide relevant information to natural resource managers seeking to enhance conservation efforts.

Water management, restoration efforts, and climate change are all predicted to contribute to hydrologic changes in the future [120, 121]. Shifts in environmental conditions can affect important primers and cues that influence the movements and reproductive success of migratory species [9, 100]. In Florida, analysis of long-term precipitation records suggests a shortening of the wet season, and the historic bi-modal summer rainfall patterns may become unimodal [70]. This may alter migration timing and the duration of the spawning season for species like Snook. Sea level rise threatens to increase salinities in what are currently freshwater habitats of the Everglades. Water management practices that increase freshwater inputs from the north to keep salinity at bay could increase water depth and marsh flooding duration [120]. Conversely, shifts in atmospheric teleconnections (i.e., the AMO) that decrease future rainfall could result in shallower marsh depths, and decrease the abundance of freshwater prey [64, 107, 121]. Water management can mitigate potential climate impacts, and as conditions shift under climatic change, understanding how these changes will affect animal migration and the consequences for population trends can inform conservation efforts aimed at preserving valuable fisheries. Providing natural resource managers with quantitative descriptions of both the primers and cues that influence spawning migrations can assist in maximizing reproduction and recruitment for ecologically, economically, and culturally important species, such as the Common Snook.

## Supplementary Information

**Additional file 1.** Contains supplementary figures and tables and includes plotted hydrographs of water levels at Bottle Creek and MO215 monitoring stations for each year of the study (**Fig. S1**), descriptions of all model variables considered and selected for use in final models for hypothesis 1 (**Table S1**), descriptions of all model variables used in models for hypothesis 2 (**Table S2**), the number of new acoustic tags deployed, the number of individual fish detected, and the number of fish migrating for each year of the study (**Table S3**), and additional data showing the number of fish detected migrating each month for each year of the study (**Table S4**).

## Data Availability

The acoustic telemetry datasets generated and analyzed during the current study are available through the Florida Coastal Everglades Long Term Ecological Research Program under the Environmental Data Initiative [122].

## Abbreviations

AIC: Akaike information criterion
AMO: Atlantic multidecadal oscillation
ENP: Everglades National Park
GLMM: Generalized linear mixed model
NAVD 88: North American Vertical Datum of 1988
PSU: Practical salinity unit
SPR: Spawning potential ratio
TL: Total length
