# Supplementary material for: Primed and cued: long-term acoustic telemetry links interannual and seasonal variations in freshwater flows to the spawning migrations of Common Snook in the Florida Everglades
Source: Mov Ecol. 2022 Nov 13;10:48. doi: 10.1186/s40462-022-00350-5 (PMC9655820; doi:10.1186/s40462-022-00350-5)
Supplement: Supplementary file 1 — Additional file 1. Contains supplementary figures and tables and includes plotted hydrographs of water levels at Bottle Creek and MO215 monitoring stations for each year of the study (Fig. S1), descriptions of all model variables considered and selected for use in final models for hypothesis 1 (Table S1), descriptions of all model variables used in models for hypothesis 2 (Table S2), the number of new acoustic tags deployed, the number of individual fish detected, and the number of fish migrating for each year of the study (Table S3), and additional data showing the number of fish detected migrating each month for each year of the study (Table S4). [file 40462_2022_350_MOESM1_ESM.pdf]

Additional File 1
Massie et al.
Primed and cued: long-term acoustic telemetry links interannual and seasonal variations in freshwater flows to the spawning migrations of Common Snook in the Florida Everglades
Movement Ecology

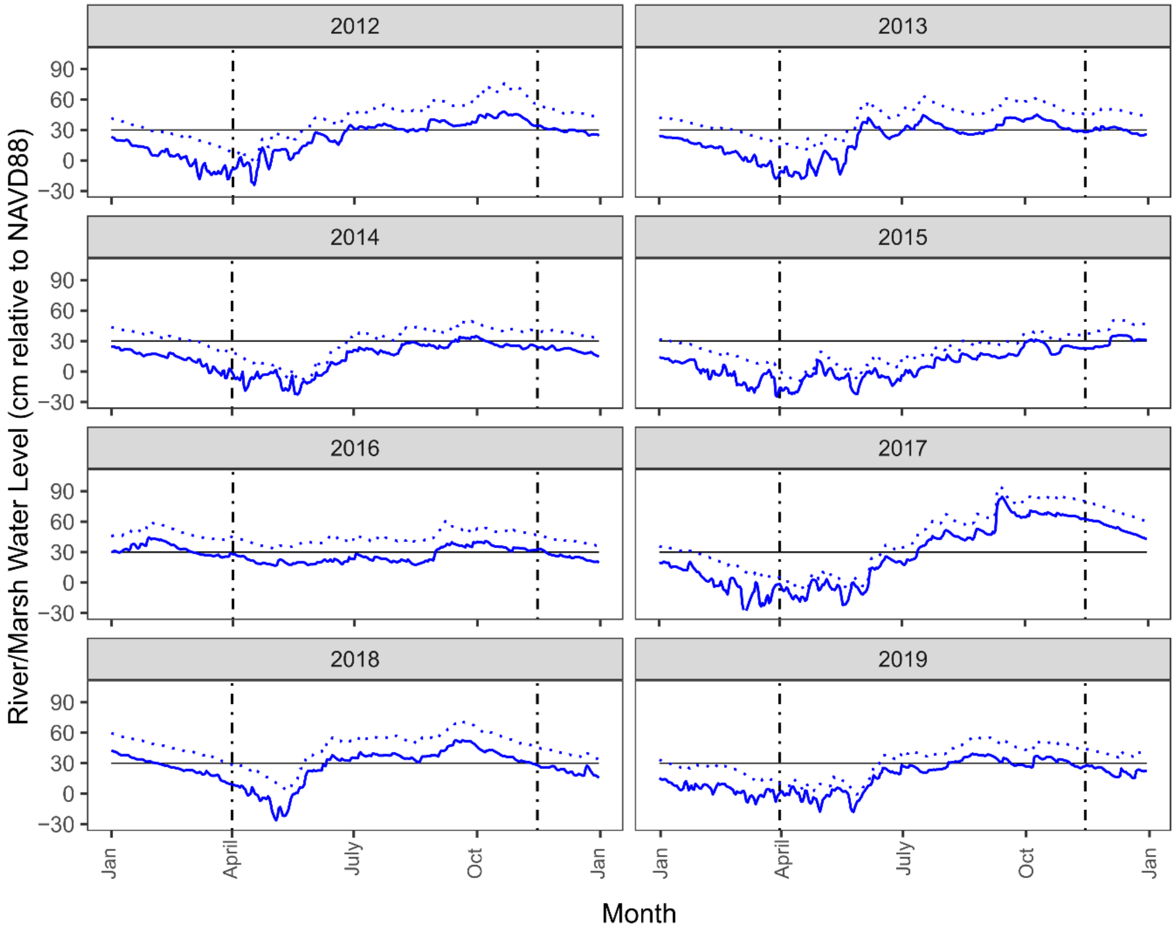

Fig. S1: Annual hydrographs for two monitoring stations in the Shark River from 2012 to 2019 illustrating the high degree of interannual variability in flow timing and magnitude. Solid blue lines show water levels at Bottle Creek in the upper river and dotted lines reflect water level in the adjacent freshwater marshes at station MO215 (see Fig. 2b for station locations). Vertical hashed lines indicate the beginning and end of the spawning season (April 1-Nov 15) used in statistical models, and the solid horizontal line shows the 30 cm water depth corresponding to the beginning of the wet/dry transitional period and marsh prey concentration in the river channels.

Table S1: Variables considered and selected for use in GLMMs for hypothesis 1, that interannual differences in hydrologic conditions preceding the spawning season (migratory primers) contribute to the intensity of the Common Snook migratory response at a broad temporal scale. Model variables are organized by hypothesized drivers, and bolded variables indicate those included in our global model, which were selected from collinear variables using lowest AIC.

Hypothesized Driver	Model Variable	Description
Flow Dynamics	Maximum Water Level	Maximum dry season water level in the upper river (cm)
	Minimum Water Level	Minimum dry season water level in the upper river (cm)
	Water Level Range	Dry season water level range in the upper river (Max - Min)
	Drydown Duration	Number of days during the spring drydown preceding spawning season where marsh water level at station MO215 was < 30 cm
Other Hydrologic/Abiotic Conditions	***Maximum Temperature***	Maximum dry season water temperature in the upper river (degrees C)
	Minimum Temperature	Minimum dry season water temperature in the upper river (degrees C)
	Temperature Range	Dry season water temperature range (Max - Min)
	Maximum Salinity	Maximum dry season salinity in the upper river (PSU)
	Minimum Salinity	Minimum dry season salinity in the upper river (PSU)
	Salinity Range	Dry season salinity range (Max - Min)
Fish Size	***Fish Size***	Total length (cm) of tagged Common Snook at the beginning of each spawning season

Table S2: Variables selected for use in GLMMs for hypothesis 2, that specific cues trigger Common Snook migration timing during the spawning season. Model variables are organized by hypothesized drivers and evaluate the relative role of flow dynamics, other hydrologic/abiotic factors, seasonality, and interannual variability in migration timing.

Hypothesized Driver	Model Variable	Description
Flow Dynamics	Water Level	Mean daily water level in cm recorded at monitoring station in the upper river at Bottle Creek
	Daily Water Level Change	Daily rate of change in water level (Current day mean - previous days mean)
Other Hydrologic/Abiotic Conditions	Temperature	Mean daily water temperature in the upper river (degrees C)
	Salinity	Mean daily salinity in the upper river (PSU)
Phenology	Photoperiod	Total hours of daylight
	Lunar Period	Numeric variable indicating the fraction of the lunar period from the full moon on that day
Interannual Variation	Year	Calendar year of spawning season

Table S3: Number of new acoustic tags deployed in the Shark River for each year of the study, the number of individual Common Snook detected during the spawning season, the number of individuals detected making a coastal migration on the acoustic array, and the annual proportion of detected fish that migrated. †Note: a portion of individuals were detected in multiple years, and summed annual detections include repeat observations for some fish.

Year	# New Tags Deployed	# Snook Detected	# Snook Migrating	Proportion Migrating
2012	44	34	18	0.53
2013	17	32	8	0.25
2014	20	36	12	0.33
2015	21	38	20	0.53
2016	55	47	5	0.11
2017	14	34	15	0.44
2018	21	34	6	0.18
2019	14	42	19	0.45
Total	206	297†	103	

Table S4: Number of Common Snook migrations detected by month and year from 2012-2019. Detections reflect individuals making directed movements from the upper river/Tarpon Bay into the lower river during the spawning season (April 1 – Nov. 15). Numbers include individuals that were detected migrating on multiple days, or those making repeat migrations within a spawning season. The proportion of total detected migrations for all years of the study are summarized by month.

Year	April	May	June	July	Aug	Sept	Oct	Nov
2012	5	5	4	0	7	1	3	3
2013	1	2	4	0	0	1	1	2
2014	2	3	5	3	4	3	2	3
2015	15	2	3	3	1	1	3	0
2016	0	2	3	1	0	0	0	0
2017	0	3	4	0	0	12	1	1
2018	0	3	2	1	1	0	1	0
2019	5	5	9	5	3	2	3	2
Proportion	0.17	0.16	0.21	0.08	0.1	0.12	0.09	0.07
